# Editorial Department

**Published:** 1856-02

**Authors:** 


Dr. W. J. Holt, a young American surgeon, engaged in the Russian service in the Crimea, is winning golden opinions from the Northern Bear, by the manner in which he is treating his wounded soldiers—and the golden opinions of the Emperor are almost equal to the ready cash.—*Western Journal of Medicine and Surgery.*

